# Silent Uterine Rupture with the Use of Misoprostol for Second Trimester Termination of Pregnancy : A Case Report

**DOI:** 10.1155/2011/584652

**Published:** 2011-04-19

**Authors:** Martin Cuellar Torriente

**Affiliations:** National District Hospital, P.O. Box 20147, Willows, Bloemfontein, Free State 9320, South Africa

## Abstract

Uterine rupture is an uncommon, but a life-threatening, complication following second trimester medical termination of pregnancy (TOP). The reported cases have been in both the scarred and unscarred uterus (Rajesh et al. 2002, Drey et al. 2006, and Dickinson). A 27-year-old with two previous deliveries, no previous caesarean section, no history of induced abortions, and no gynaecological operations. She presented with amenorrhoea, and according to her last normal menstruation, she was 10 weeks and 5 days. Ultrasound was done, and it reported 16 weeks and 5 days. She asked for TOP. According to the clinic's protocol, misoprostol 800 mcg (4 tabs) were given to be used vaginally as a loading dose and another three to be taken orally after that. In the following day when she attended the clinic for follow up, a manual vacuum aspiration (MVA). A manual vacuum aspiration was indicated as an incomplete abortion. During the procedure, a uterine rupture was found in the uterine lower segment. A laparotomy was done and a lineal uterine rupture was found and sutured. The patient had a good postoperative recovery and was discharged from hospital after four days. The clinician dealing with second trimester terminations should be aware of the possibility of having a uterine rupture, especially in patients with a uterine scar in order to make an early diagnosis.

## 1. Introduction

Uterine rupture is an uncommon, but a life-threatening complication following second trimester medical termination of pregnancy (TOP). The reported cases have been in both the scarred and unscarred uterus [1–3]. The occurrence is about 0.2% in the intact uterus and 3.8 to 4.3% in the scarred uterus [1, 2].

## 2. Case Report

A 27 year-old woman with two previous deliveries, no history of previous caesarean section, no induced abortions, and no gynaecological operations, presented with amenorrhoea seeking for TOP in her third pregnancy. She had no pregnancy symptoms and no history of contraceptive use. According to her last normal menstrual period, her gestational age was ten weeks and six days. 

On physical examination, blood pressure was 110/70, and pulse was 76 beats per minute. Symphisis fundal height was 15 cm. An ultrasound scan was done. It reported 16 weeks and 5 days (Biparietal diameter, femur length, and abdominal circumference).

The patient was counselled for termination of pregnancy; the methods, side effects, and complications were explained. Alternative options were also discussed. She opted for TOP. 

Based on the Choice of Termination of Pregnancy Act, 1996 (CTOP, Act 1996), her request was accepted. According to the clinic's protocol, seven misoprostol tablets (Cytotec 200 mcg) were issued to the patient with the full instructions on how to be used at home. Four tablets (800 mcg) to be inserted deep in vagina as a loading dose. Three hours later she was instructed to use one tablet every 2 hours orally for three doses.

She inserted the tablets at 22:00 that night. She aborted at 07:00 the following morning while she was coming to the clinic for follow up.

On examination, a contracted, not tender uterus, 6 cm above the pelvis brim, was found, as well as a soft abdomen. On vaginal examination, she presented with a minor bleeding and an opened cervix. A manual vacuum aspiration indicated that she had an incomplete abortion. During the introduction of the cannulae, a uterine rupture was diagnosed in the lower segment.  The Hb at that stage was 7.6 g/dL. Two units of blood were ordered.The patient was prepared and booked for theatre immediately for an exploratory laparotomy. During the operation, 50 mL of blood was found in the abdomen with a linear rupture of 6 cm in the lower segment of the uterus. This was sutured in one layer, covering with the visceral peritoneum without complications. The patient was given antibiotics as per protocol (Amoxicillin and Methronidazole) and had a good postoperative recovery. She was discharged four days later.

## 3. Discussion

 Medical abortion was started in the late eighties, becoming more widely used in the late nineties with the mifepristone and misoprostol being the most used. It came as an alternative for the dilation and curettage which caused more complications, resulting in 50,000–100,000 maternal deaths every year [4, 5]. 

Misoprostol alone for termination of pregnancy (TOP) was described for the first time in 1994. It has been used widely for TOP in the normal uterus [4, 5]. Other prostaglandins (gemeprost, sulprostone) have been used but those are more expensive and have not been used widely. Methotrexate has also been used, but the gynaecologists are not comfortable with it due to its teratogenicity. Extra-amniotic methods were used with prostaglandin instilled through an intracervical catheter [3]. Mifepristone prostaglandin has been shown to be safe and effective, with a success rate of 92–96% [6, 7]. Misoprostol has been used for TOP in different ways, vaginal, oral, and in combination of the two routes in the first and second trimester [8]. The mortality and morbidity of the second trimester is greater than that of the first trimester termination. Abortion-related morbidity and mortality increase significantly as pregnancy advances with a sharp rise in the rate of severe complications in induced abortion after 14 weeks of pregnancy [9, 10]. Women present with varying reasons for the delay in presenting, being logistical, social, or economic. Uterine rupture with the use of misoprostol has been reported more frequently in multiparous women and in women with uterine scars. It is more often observed at term than in the second trimester [3]. The rate of caesarean births has been on the rise, it has lead to and increasing numbers of women with a uterine scar seeking TOP [3–7]. The Reproductive Health Clinic at National District Hospital in Bloemfontein has been working for more than a decade dealing with termination of pregnancy. During that period, misoprostol alone has been used, combining the vaginal and oral route (800 mcg vaginally as a loading dose and 600 mcg orally in three divided doses) with a high success rate and no known previous complication. The clinician dealing with second trimester terminations should be aware of the possibility of having a uterine rupture, especially in patients with a uterine scar in order to make an early diagnosis.
